# Endometrial thickness evaluation with ultrasonography assessment among normal and endometrial cancer across multi-ethnic Indonesian women

**DOI:** 10.3389/frph.2026.1826142

**Published:** 2026-07-23

**Authors:** Andi Darma Putra, Aldi Tamara Rahman, Naufal Syafiq Darmawan, Lasmini Syariatin, Firda Puspita

**Affiliations:** 1 Division of Gynecology-Oncology, Department of Obstetrics and Gynecology, Faculty of Medicine, Universitas Indonesia, Cipto Mangunkusumo Hospital, Central Jakarta, Indonesia; 2 Dopamine Science Institute, Depok, Indonesia; 3 Ovarian, Tubal, and Peritoneal Malignancy Research Unit, Department of Obstetrics and Gynecology, Faculty of Medicine, Cipto Mangunkusumo Hospital, Central Jakarta, Indonesia

**Keywords:** endometrial cancer, endometrial thickness, Indonesia, normal endometrial, ultasonography

## Abstract

**Introduction:**

Endometrial thickness is a crucial indicator for reproductive health evaluation and initial detection of abnormalities. This study aims to comprehensively characterize and evaluate the endometrium in normal and cancer of Indonesian women using ultrasonography.

**Methods:**

This retrospective investigation analyzed ultrasound data from three hospitals during 2021–2024 in women who met predefined inclusion criteria. Subjects were classified according to age, ethnicity, menopausal status, and ultrasound-based examination category.

**Results and Discussion:**

Endometrial thickness was quantified in 170 women with normal gynecological result and 82 women with histopathologically confirmed endometrial cancer. The average age of the normal group was 40.63 ± 0.91 years while the cancer group had an average age of 52.28 ± 1.33 years, significantly indicating that cancer subjects were generally of greater age. Endometrial thickness significantly decreased with advancing age, with the reduces values in women over 50 years (post-menopausal). There was a significant difference in endometrial thickness between normal and cancer cases, with optimal diagnostic cut-off values of 12.85 mm (AUC 0.89, sensitivity 0.83, specificity 0.94) for women aged ≤50 years and 10.20 mm (AUC 0.99, sensitivity 0.97, specificity 1.00) for women aged >50 years. These results underscore the robust relationship between age, menopausal status, and endometrial pathology, and support the clinical utility of ultrasound as a non-invasive method for early detection and risk profiling of endometrial abnormalities.

## 1 Introduction

The endometrium consists of epithelial cells, stromal tissue, and glandular structures, including both functional and basalis layers (1). It undergoes cyclical redevelopment during the menstrual cycle, characterized by proliferation, differentiation, and shedding during, while also provides the adequate environment for embryo implantation during the peri-ovulatory phase (2). During implantation, the endometrium facilitates early embryonic development by providing nutrients, controlling the maternal immune response, and regulating trophoblast invasion (3, 4). These processes are rigorously regulated by physical and molecular factors. Physical factors include endometrial thickness, uterine and endometrial blood flow, whereas molecular factors encompass hormonal regulation, local metabolic and vascular dynamics, and overall systemic health, all of which contribute to the preservation of reproductive viability (5–7). Endometrial thickness may serve as a physical marker for women's reproductive health, reflecting fertility potential. The endometrial dimensions, particularly its thickness, are influenced by various factors, such as age, race/ethnicity, body mass index, and follicle-stimulating hormone (FSH) concentrations (8). Further, the histological characteristics of an endometrium evolve through the pre-menopause, perimenopause, and post-menopause stages (9).

Previous research has demonstrated a positive correlation between endometrial thickness and hormonal status, including estradiol and progesterone, which may aid in assessing reproductive health and supporting pathological evaluation (10, 11). For instance, highly abnormal endometrial thickness may serve as a biomarker for the development of polycystic ovarian syndrome (PCOS) in patients (12). Endometrial thickness measurements exhibit significant variations among multiple populations, suggesting genetic, environmental, and methodological differences globally. Tsuda et al. reported a median endometrial thickness of 8.6 mm among premenopausal Japanese women (13). Davar et al. observed an average of 10 mm in premenopausal African women population, while Bakare et al. evaluated postmenopausal women, revealing an average of 2.17 mm and a prevalence of just 1.1% for thickness exceeding 5 mm (14, 15). Women with endometrial cancer showed significantly higher endometrial thickness, ranging between 15 and 16 mm (16). These values vary depending on menopausal status, cancer subtypes, and ethnical background (17–19). However, detailed evidence on endometrial thickness characteristics in the Indonesian population remain limited. Therefore, this study aims to investigate the endometrial dimensions in healthy women and those diagnosed with endometrial cancer, addressing an existing research gap. Ultrasonography presents as a favorable method due to its ability to reduces patient exposure to ionizing radiation, enables multi-sectional organ imaging, and provides convenience at a relatively low cost. In particular, transvaginal and transabdominal ultrasonography are widely used and precise diagnostic techniques, frequently applies in the evaluation of pelvic pathologies, including endometriosis (20, 21).

This study aims to characterize the endometrium of Indonesian women using ultrasonography, with a primary focus on evaluating endometrial thickness across different age groups, reproductive stages, and pathological conditions. Our findings are expected to provide valuable insights into endometrial dimensions within the Indonesian population, support more accurate diagnosis and management of reproductive health conditions, and inform the development of population-specific medical interventions. Additionally, this study establishes a novel dataset on endometrial thickness stratified by age and ethnic background in both healthy women and women with endometrial cancer.

## 2 Methods

### 2.1 Ethical clearance

This retrospective study employed patient medical records for data collection. Ethical approval was granted prior to data collection from Ethics Committee of the Faculty of Medicine, Universitas Indonesia–Cipto Mangunkusumo of National Hospital (Ethics approval code: KET-675/UN2.F1/ETIK/PPM.00.02/2025). A waiver of informed consent was authorized due to the study's retrospective approach.

### 2.2 Study design

This study comprised two retrospectively collected cohorts: women with normal endometrial findings and patients with endometrial cancer. Data for the normal cohort initially included 1,001 women who underwent ultrasonography and were subsequently filtered according to predefined inclusion and exclusion criteria before analysis. Only the eligible normal participants were then stratified into five age groups: 20–30, 31–40, 41–50, 51–60, and over 60 years, whereas endometrial cancer cases, confirmed histopathologically between 2021 and 2024, were categorized into three age groups: 41–50, 51–60, and over 60 years. The endometrium was assessed morphometrically using transvaginal ultrasonography at three centers: Cipto Mangunkusumo Hospital (Central Jakarta), Hermina Hospital Depok, and Pantai Indah Kapuk Hospital (North Jakarta). To minimize interobserver variability, all ultrasound examinations and endometrial thickness measurements were performed by a single experienced operator (first author). During the same examination, endometrial vascularity was evaluated using a 4-point ordinal scale (1 = no detectable color flow, 2 = minimal flow, 3 = moderate flow, 4 = abundant flow) based on color or power Doppler assessment of the most vascular areas of the endometrium.

### 2.3 Ultrasound data collection and validation

Ultrasonographic evaluations were conducted with the Mindray Resona 7 (V11-3HU probe; Mindray, USA) and the Voluson Expert 22 (Convex Matrix Array Volume RM7C probe; GE Healthcare, Austria). Both systems employ micro-convex transducers tailored for intracavitary gynecological imaging and include two-dimensional, Doppler, elastography, and volumetric imaging methods. Measurements were acquired in the longitudinal plane with a frequency range of 3–11 MHz to assess endometrial thickness. All measurements and image acquisitions were conducted according to the International Endometrial Tumor Analysis (IETA) consensus guidelines. Patients fulfilling the inclusion criteria, namely normal gynecological status and absence of relevant comorbidities for the normal cohort and histologically confirmed endometrial cancer for the cancer cohort, were included, while those with significant comorbidities or incomplete or biased records were excluded and classified as dropouts. None of the enrolled patients were receiving hormonal therapy at the time of ultrasonographic evaluation. The final dataset comprised mean age at premenopause, menopause, and postmenopause; average endometrial thickness in normal women and in those with endometrial cancer; distribution of endometrial thickness by ethnicity; ultrasonographic images; and the relationship between depth of myometrial invasion and the color score.

### 2.4 Exclusion and inclusion criteria

Eligibility criteria for women with a normal endometrium:

1. Exclusively participants free from detectable abnormal results during gynecological assessment were recruited. This was defined by the absence of ultrasound-based or clinical evidence of gynecological abnormalities involving the female reproductive tract, including endometrial hyperplasia, endometrial polyps, uterine fibroids (leiomyomas), adenomyosis, ovarian cysts or solid ovarian lesions, polycystic ovary syndrome (PCOS), and abnormal uterine bleeding patterns suggestive of underlying uterine pathology.
2. Participants showed no recorded previous history of pathological gynecological disorder.
3. Participants showed no evidence of confirmed genetic abnormalities that might affect endometrial morphology or thickness. In addition, participants with known genetic abnormalities potentially affecting endometrial morphology or thickness, such as Lynch syndrome, Cowden syndrome, or other hereditary cancer predisposition syndromes involving the endometrium, were excluded from the study.
4. Every recruited participant had completed a standard gynecological assessment before study entry, with findings compatible with the absence of the above-mentioned pathologies.

Exclusion criteria for the normal endometrium group:

1. Participants with a verified diagnosis of any gynecological disease were remove from the study.
2. Participants with a previous history of pathological gynecological disease were not suitable for inclusion.
3. Participants with hereditary disorders known to affect endometrial morphology, endometrial thickness, or endometrial cancer risk were excluded. This included individuals with inherited conditions such as Lynch syndrome, Cowden syndrome, and other hereditary cancer predisposition syndromes associated with endometrial involvement.

Inclusion criteria for the endometrial malignancy group:

1. Exclusively women with a biopsy-proven diagnosis of initial endometrial carcinoma were included.
2. Female patients with a confirmed diagnosis established through laparotomy or hysterectomy, with subsequent histopathological examination and verified results.
3. Eligible participants had received transvaginal or transabdominal gynecologic ultrasound with endometrial thickness able to be accurately quantified.
4. Comprehensive clinical records concerning participants age, menopausal state, body mass index, and ethnic origin was necessary for enrollment.

Exclusion criteria for the endometrial malignancy group:

Endometrial cancer cases were omitted from analysis when:

1. Clinical records or ultrasound data not available, partial, or unreliable (such as lack of a robust thickness of endometrium).
2. The histopathology report was unclear, non-definitive, or showed a lesion rather than primary endometrial carcinoma.
3. A prior history of uterine surgery or pelvic irradiation had significantly modified endometrial anatomy, thus impairing the reliability of endometrial thickness evaluation.

### 2.5 Statistical analysis

Descriptive statistics were performed using IBM SPSS Statistics for Windows, version 29.0 (Armonk, NY: IBM Corp). Prior to group comparisons, data were tested for normality and homogeneity of variances. For variables fulfilling assumptions of normality and homogeneity, differences among groups were analyzed using one-way analysis of variance (ANOVA), followed by Tukey's *post hoc* test for pairwise comparisons when overall significance was observed (*p* < 0.05). For variables that did not satisfy parametric assumptions, the Kruskal–Wallis test was applied, followed by Mann–Whitney *U* tests for pairwise comparisons. A two-tailed *p*-value < 0.05 was considered statistically significant. Multivariable binary logistic regression was performed to evaluate the association between endometrial thickness and endometrial cancer after adjustment for age and body mass index (BMI). Receiver operating characteristic (ROC) curve analysis was performed to determine the optimal cutoff value for distinguishing normal from cancer endometrial thickness. Diagnostic performance was evaluated based on the area under the curve (AUC), and the optimal threshold was selected using the Youden index.

## 3 Results

**Figure 1:**
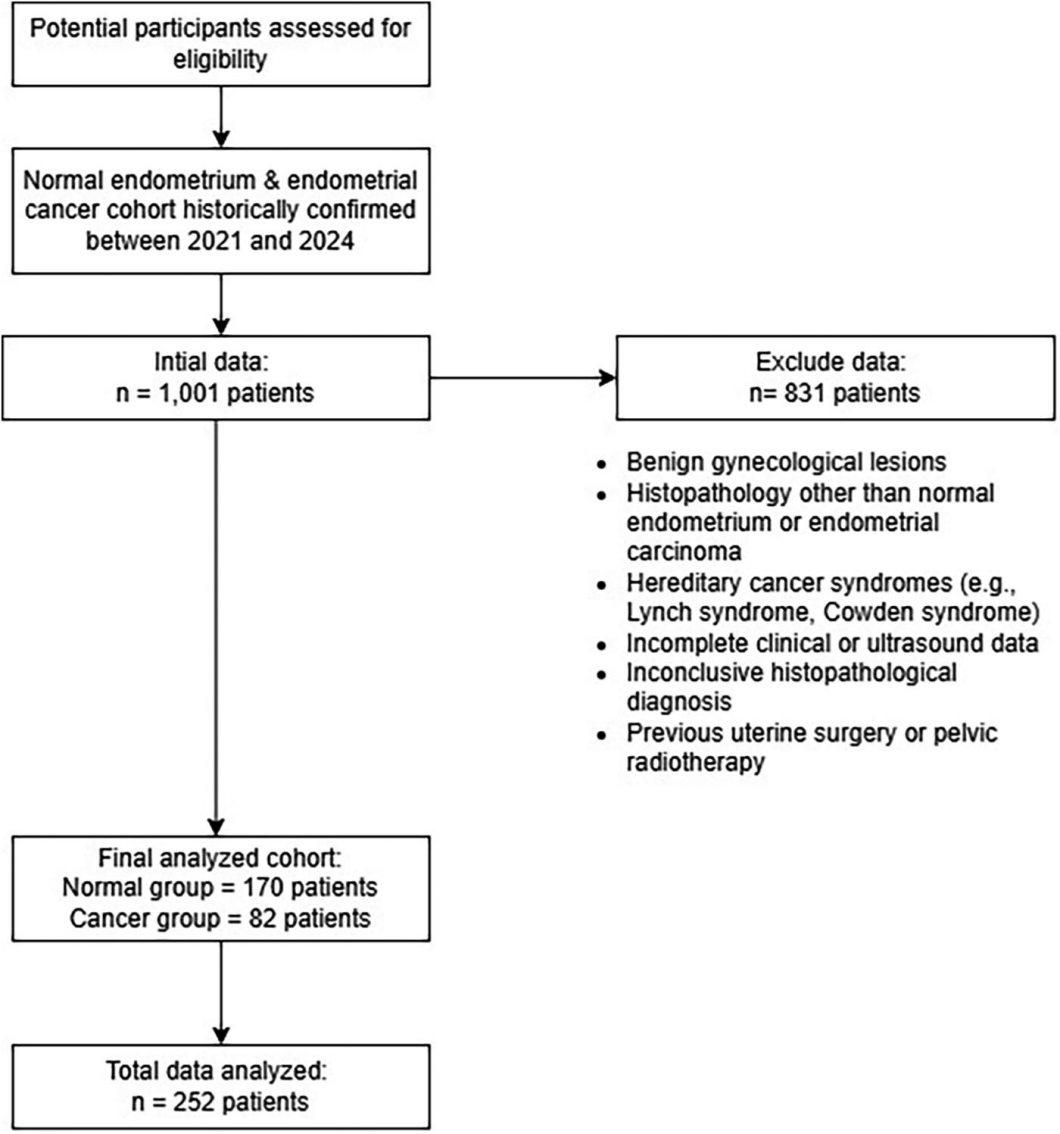
Participant selection flow diagram showing the screening, eligibility assessment, and final inclusion of subjects in the endometrial cohort analysis.

As shown in Figure 1, endometrial thickness was evaluated in a cohort of 170 women of various ages and ethnic backgrounds who underwent ultrasonographic examination and were confirmed to have normal findings. In addition, endometrial thickness data were collected from 82 patients with histologically confirmed endometrial cancer. As shown in Table 1, the mean age of women in the normal group was 40.63 ± 0.91 years (95% CI 38.85–42.42), which was significantly lower than in the endometrial cancer group (52.28 ± 1.33 years; 95% CI 49.60–54.95). The mean age at menopause in the normal group was 52.50 ± 0.77 years (95% CI 50.66–54.34), which differed significantly from the endometrial cancer group (50.02 ± 0.56 years; 95% CI 48.83–51.23). In contrast, the interval between menopause and ultrasonographic examination was 6.33 ± 1.47 years (95% CI 2.93–9.73) in the normal group and did not differ significantly from the 9.61 ± 1.04 years (95% CI 7.50–11.73) observed in the endometrial cancer group. The mean body mass index (BMI) was significantly higher in endometrial cancer group than in the normal group (23.60; *p* < 0.05).

**Table 1:** Baseline characteristic of participants.

Characteristic	Normal group, *n* *=* 170	EC group, *n* = 82	*p*-value
Age	40.63 (5–77)	52.28 (29–80)	<0.001
Age at menopause	52.50 (50–56)	50.02 (40–59)	0.020
Year since menopause	6.33 (2–15)	9.61 (2–30)	0.117
Body mass index (BMI)	23.60 (15.63–34.21)	27.35 (14.71–43.72)	<0.001

Data are given as mean (range).

**Figure 2:**
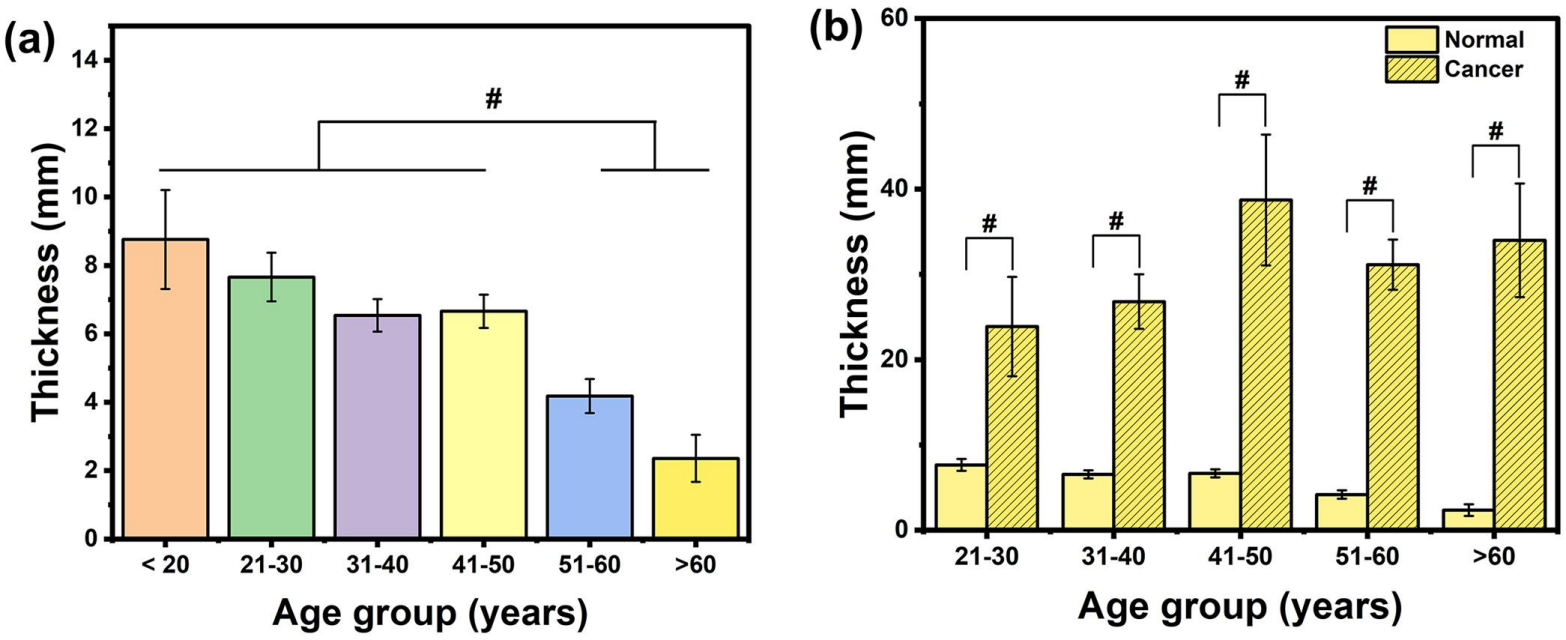
Endometrial thickness: **(a)** normal group; **(b)** comparison between the normal group and the endometrial cancer group. The symbol # indicates a statistically significant difference according to the Mann–Whitney *U* test.

**Figure 3:**
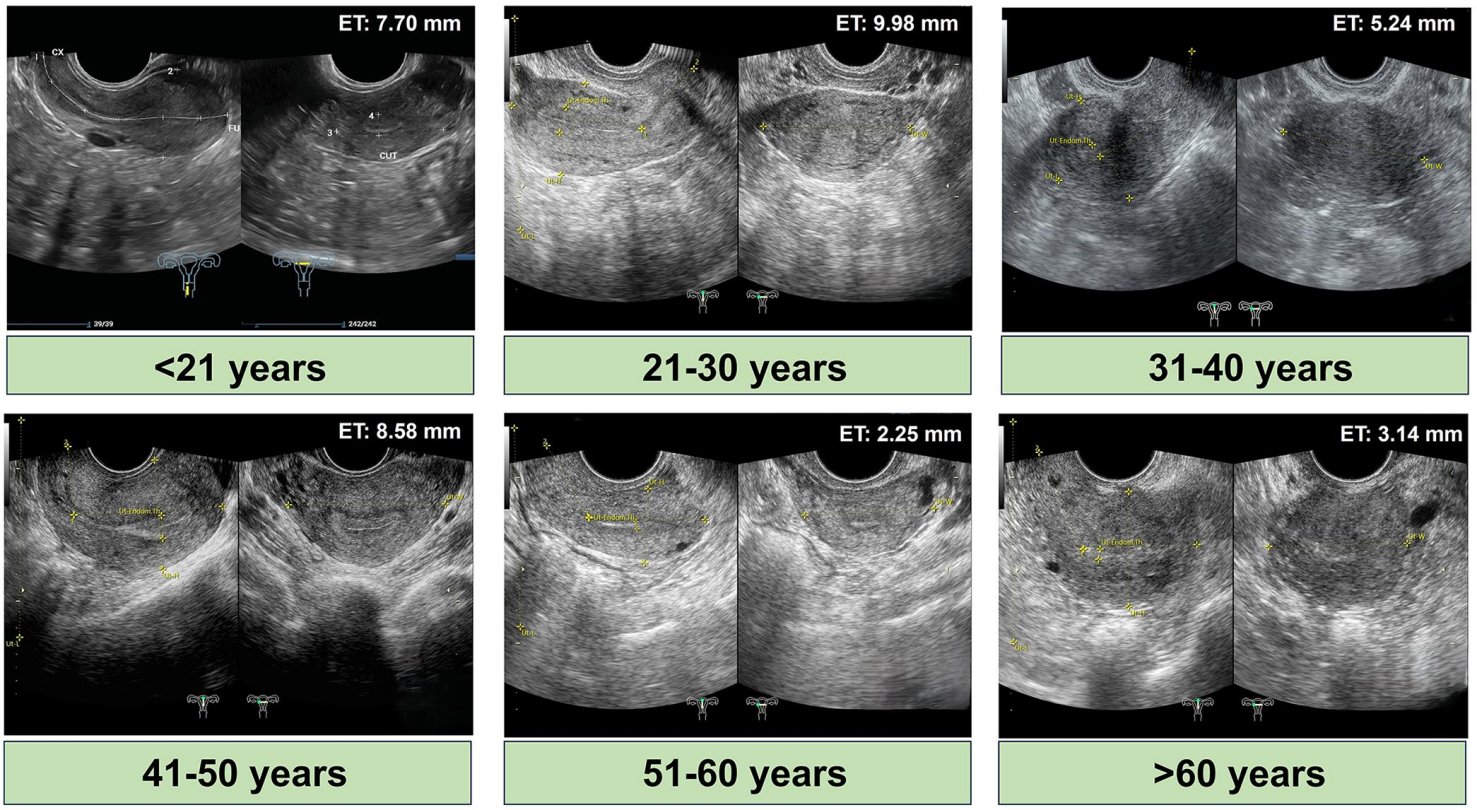
Endometrial thickness in normal conditions across different age groups, ET: endometrial thickness.

Endometrial thickness in the normal group showed a clear age-dependent decline. Mean values were 8.76 ± 1.45 mm (95% CI: 4.74–12.78) in women younger than 20 years, 7.66 ± 0.71 mm (95% CI: 6.21–9.11) in those aged 21–30 years, 6.54 ± 0.45 mm (95% CI: 5.59–7.50) in the 31–40-year group, 6.66 ± 0.48 mm (95% CI: 5.69–7.64) in women aged 41–50 years, 4.18 ± 0.50 mm (95% CI: 3.15–5.21) in those aged 51–60 years, and 2.36 ± 0.68 mm (95% CI: 0.60–4.13) in women older than 60 years (Figures 2a, 3). A global difference in endometrial thickness across age categories was confirmed by the Kruskal–Wallis test (H = 27.83, df = 5, *p* < 0.05). *Post-hoc* Mann–Whitney tests showed a significant reduction in endometrial thickness in women aged 51–60 years compared with all younger age groups (<51 years; all adjusted *p* < 0.05; Supplementary Table S2), indicating a marked thinning of the endometrium after the age of 50 years (Figures 2a, 3). Notably, women under 20 years of age exhibited the greatest endometrial thickness compared with all other age groups.

**Figure 4:**
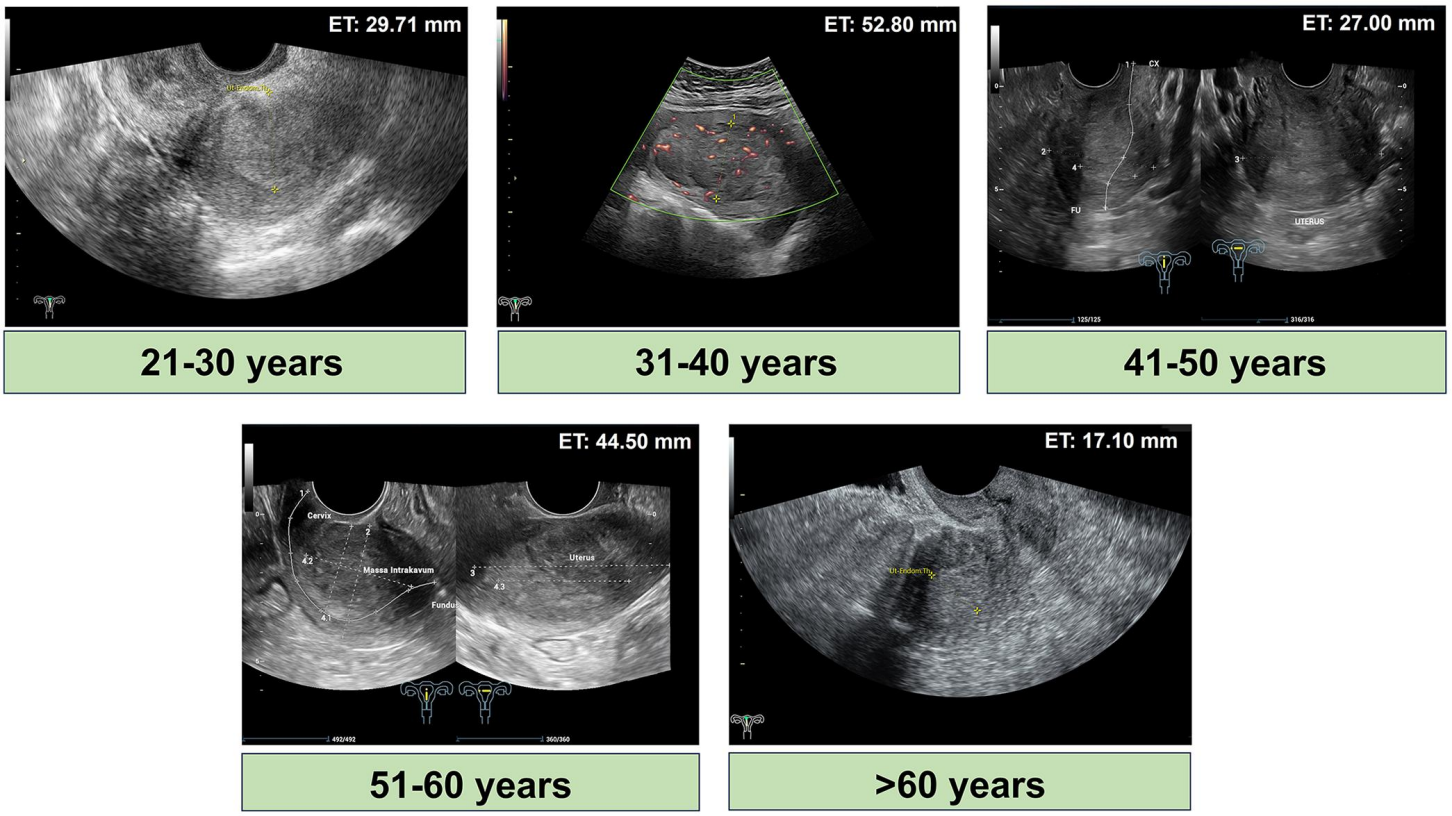
Endometrial thickness in pathological conditions (endometrial cancer) across different age groups, ET: endometrial thickness.

**Figure 5:**
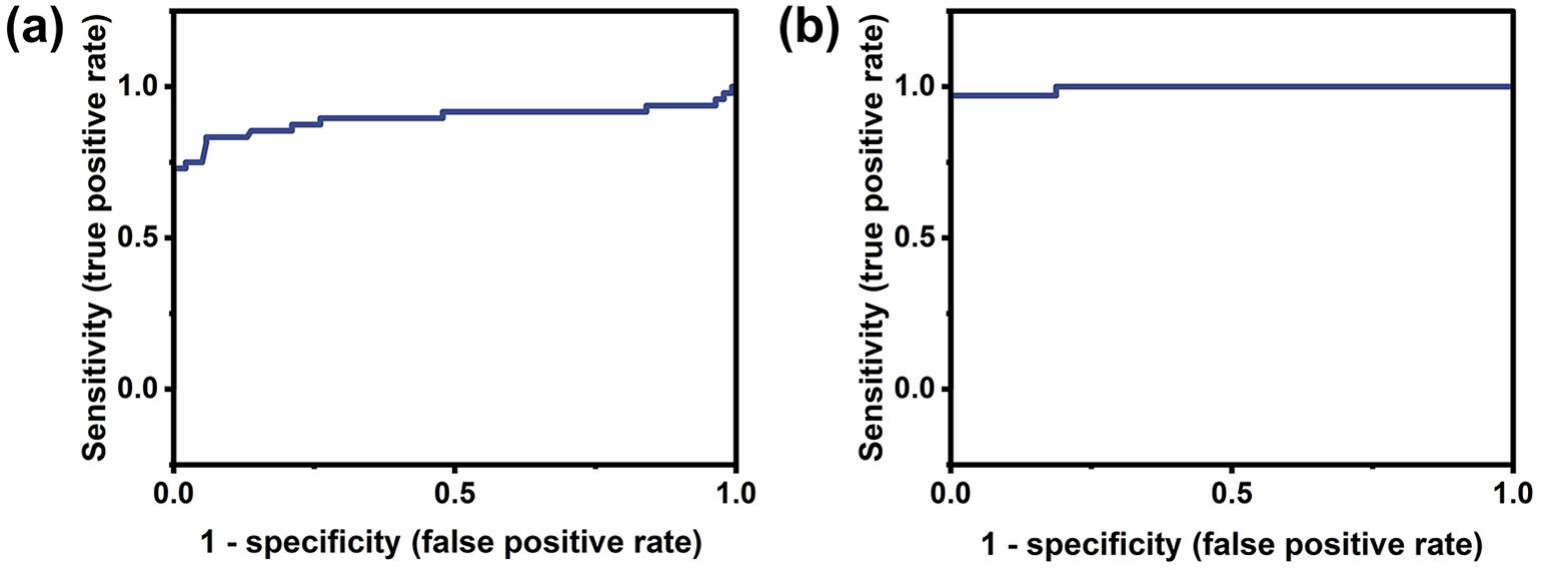
ROC curve of endometrial thickness for normal versus cancer endometrium. **(a)** ≤50 years and **(b)** >50 years.

This study further compared endometrial thickness between women in the normal group and those with endometrial cancer. As shown in Figures 2b, 4, within comparable age groups, patients with cancer exhibited markedly increased endometrial thickness, with statistically significant differences in all strata (*p* < 0.05 by Mann–Whitney test; complete U statistics and *p* values for each age group are presented in Supplementary Table S3). The mean values were 23.88 ± 5.83 mm (95% CI 7.69–40.06) at 21–30 years, 26.81 ± 3.19 mm (95% CI 20.27–33.35) at 31–40 years, 38.71 ± 7.67 mm (95% CI: 22.24–55.17) at 41–50 years, 31.13 ± 2.95 mm (95% CI 24.98–37.28) at 51–60 years, and 34.00 ± 6.66 mm (95% CI 19.49–48.52) in women older than 60 years. These findings indicate that, in the malignant condition, endometrial thickness far exceeds the average values observed under normal physiological conditions (22). Consistent with the multivariable analysis presented in Table 2, both endometrial thickness and age were identified as independent predictors of endometrial cancer meanwhile BMI was not independently associated with endometrial cancer. Furthermore, our study demonstrated that the optimal endometrial thickness cutoff for diagnosing endometrial cancer differed between women aged ≤50 years and those aged >50 years. Furthermore, receiver operating characteristic (ROC) curve analysis (Table 3 and Figure 5) demonstrated age-dependent diagnostic thresholds. The optimal cutoff value was 12.85 mm for women aged ≤50 years (predominantly premenopausal) and 10.20 mm for women aged >50 years (predominantly peri- or postmenopausal, with or without abnormal uterine bleeding).

**Table 2:** Independent predictor of endometrial cancer identified by multivariable analysis.

Variable	Unstandardized regression coefficient (B)	Standard error (SE)	Adjusted OR	95% CI	*p*-value
Endometrial thickness	0.406	0.082	1.500	1.277–1.762	<0.001
Age	0.080	0.034	1.084	1.014–1.158	0.017
BMI	0.100	0.080	1.105	0.946–1.292	0.208

**Table 3:** Age-specific diagnostic performance of endometrial thickness for endometrial cancer.

Group	Normal (n)	Cancer (n)	AUC (95% CI)	ET cutoff (mm)	Sensitivity	Specificity
≤50 years	138	48	0.89 (0.82–0.97)	12.85	0.83	0.94
>50 years	32	34	0.99 (0.98–1.00)	10.20	0.97	1.00

**Figure 6:**
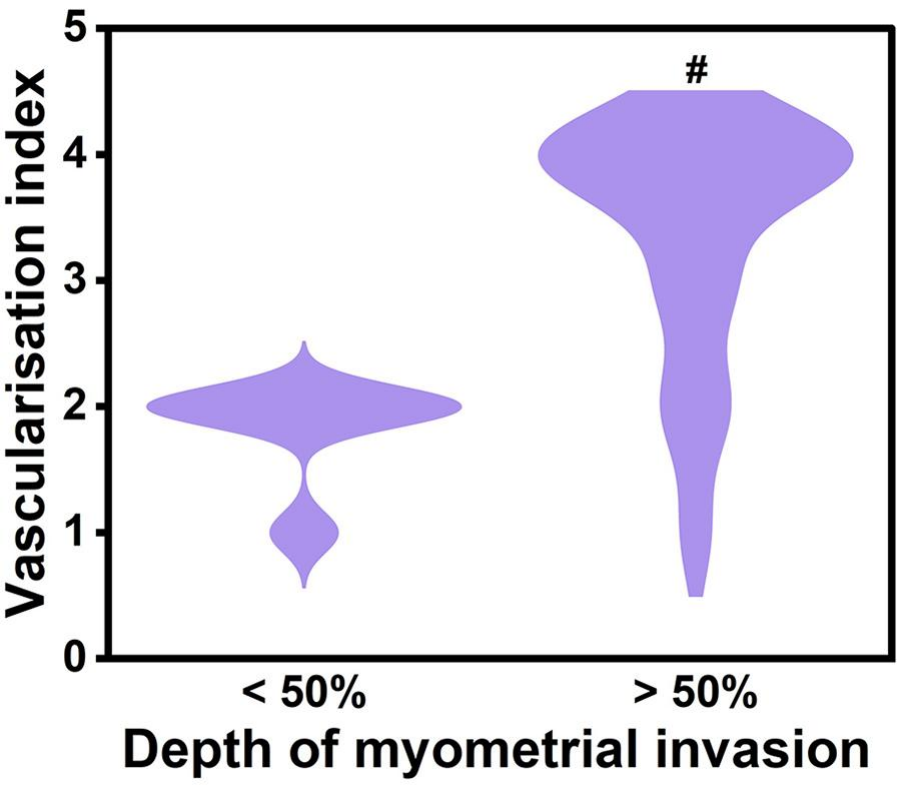
Association between depth of myometrial invasion and color Doppler score. The symbol # indicates a statistically significant difference according to the Mann–Whitney *U* test.

Moreover, most patients in the cancer group presented with myometrial invasion, a key parameter for predicting the risk of metastasis to pelvic and/or para-aortic lymph nodes (23). As illustrated in Figure 6, there was a significant difference in the color score between tumors with myometrial invasion <50% and those with invasion >50%, reflecting increased vascular density in more deeply invasive lesions. In addition, the group with invasion depth <50% showed a narrower distribution of color score compared with the >50% group, suggesting more homogeneous vascularization patterns in tumors with superficial myometrial invasion.

**Figure 7:**
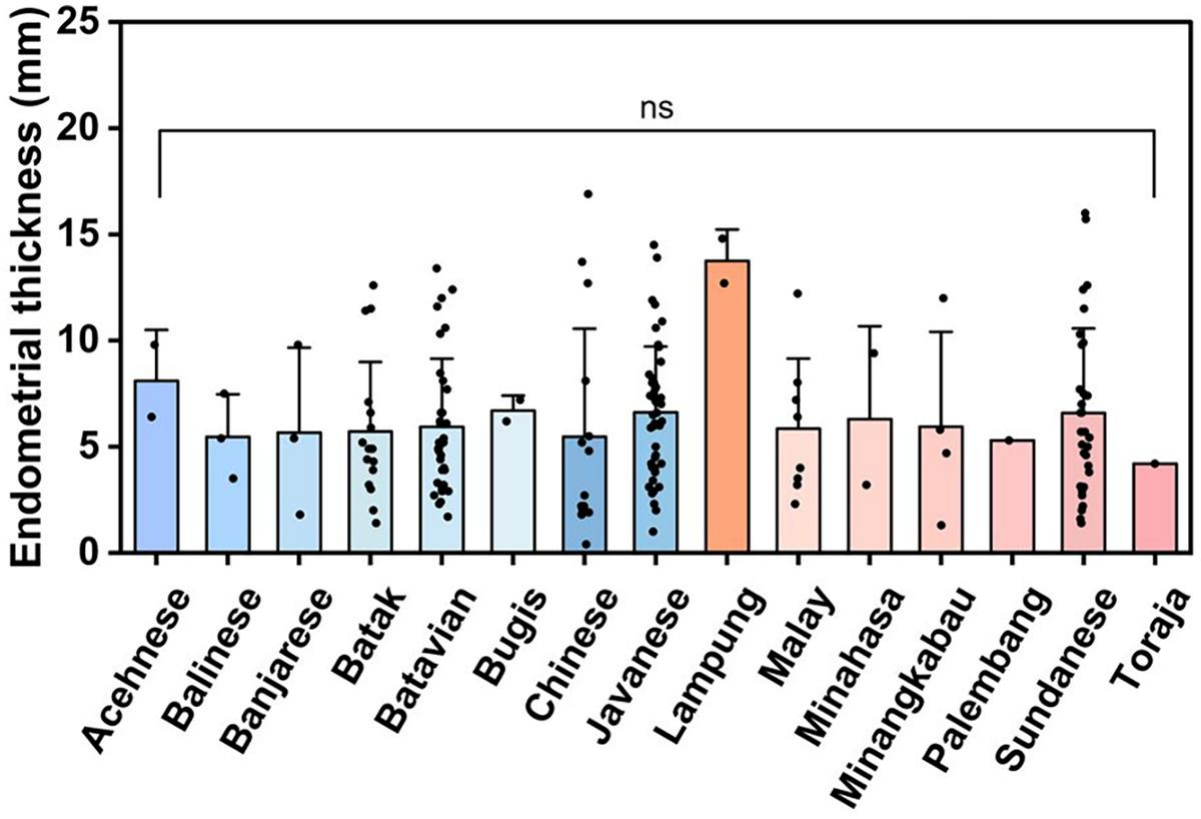
Distribution of normal endometrium thickness based on ethnicity in Indonesian women. No statistically differences were observed among groups (*p* > 0.05; according to Kruskal–Wallis test).

Normal endometrial thickness in this Indonesian cohort varied modestly across ethnic groups but remained within a similar physiological range (Figure 7). The mid-range values for endometrial thickness across ethnicities were generally between 4.2 and 8.1 mm, with overall values spanning 2.7–13.75 mm, indicating a clinically acceptable interval for this population. Kruskal–Wallis test demonstrated no significant differences in endometrial thickness among ethnic groups (H = 13.821, *p* = 0.463). These findings support the use of a common reference range for normal endometrial thickness in Indonesian women, while acknowledging minor ethnic variation. Detailed numerical data and sample sizes are presented in Supplementary Tables S1–S3.

## 4 Discussion

The normal range of endometrial thickness is a critical reference for identifying potential pathological conditions, including hyperplasia, polyps, and malignancy (20). In addition, this study reports endometrial thickness measurements in patients with histologically confirmed endometrial cancer, thereby quantitatively demonstrating how deviations from normal thickness are associated with an increased risk of malignancy (16). Endometrial thickness changes across life course of a woman and tends to decline after reproductive years, with postmenopausal values generally remaining below 3 mm (20). Moreover, endometrial thickness is also influenced by several factors, including ethnicity and demographic characteristics (8). The present study includes Indonesian women from diverse ethnic and racial backgrounds, providing an important population-specific reference for interpreting endometrial thickness in this setting.

The results indicate a significant reduction in endometrial thickness in normal women aged 51 years (Figures 2, 3). This observation aligns with the mean age at menopause identified in our cohort, as presented in Table 1. After menopause, the decline in ovarian follicles, which occurs over a woman's reproductive life, results in a marked decrease in estrogen production (24, 25). The reduction in estrogen levels is a recognized factor in endometrial atrophy, due to estrogen's critical function in promoting endometrial cell growth and proliferation (26). Furthermore, the relationship between estrogen and vascular endothelial growth factor (VEGF) indicates that the decline in ovarian estrogen during menopause leads to diminished endometrial vascularization, as demonstrated by the reduced formation of blood vessels within the endometrial tissue. As a result, the endometrium exhibits decreased activity, reduced glandular structures, increased fibrous tissue, and a gradual thinning (27, 28).

In the context of endometrial cancer, we found that endometrial thickness and age are independent parameters for assessing the likelihood of malignancy in patients. This result is consistent with the study by Schramm et al. (2017), which reported an association between endometrial cancer, age, and endometrial thickness, but no significant association with obesity or related conditions such as diabetes and hypertension, possibly due to the disproportionately high proportion of overweight or obese patients in their preselected cohort (29). Similarly, Wu et al. (2025) reported no statistically significant association between endometrial cancer and BMI, which they attributed to the high prevalence of overweight and obesity among endometrial cancer cases in developing countries and to differences in central obesity patterns in Asian populations, which were the focus of their study (30). Taken together, these findings suggest that, after accounting for age and endometrial thickness, BMI may not contribute additional independent information regarding the risk of endometrial malignancy in this clinical context. Biologically, endometrial thickness directly reflects the structural expression of local endometrial proliferation and architectural distortion, which are characteristic features of hyperplasia and malignant transformation (31). This relationship explains why endometrial thickness remains independently associated with endometrial cancer in multivariable models. In parallel, age captures the cumulative duration of hormonal cycling, repeated episodes of endometrial regeneration, and the time-dependent accumulation of genetic and epigenetic alterations in endometrial cells, all of which contribute to an increased intrinsic susceptibility to malignant change (32).

Our endometrial thickness cutoff threshold for pre-menopausal women is consistent with the recommendations of the Royal Australian and New Zealand College of Radiologists and the Cancer Australia National Centre for Gynaecological Cancers, which advise endometrial biopsy in premenopausal women when the endometrial thickness exceeds 12 mm (33). In women aged >50 years, our finding through ROC analysis yielded an optimal endometrial thickness cutoff of 10.20 mm. This value is not intended to replace the lower 4–5 mm threshold recommended by current guideline for ruling out malignancy in women with postmenopausal bleeding, which maximizes sensitivity but has limited specificity. Rather, our data suggest that in this group, endometrial thickness values between approximately 4 and 10 mm are often associated with benign and premalignant lesions, whereas thickness ≥10 mm correspond to a substantially increased likelihood of invasive carcinoma (16). These findings reflect the performance of endometrial thickness as a triage tool in a broader real-world population that includes peri- and post-menopausal women, both symptomatic and asymptomatic (34).

The variation in normal endometrial thickness observed across different racial/ethnic groups in Indonesia suggests that genetic background and possibly lifestyle or environmental factors may contribute to subtle differences in endometrial morphology. These variations may affect reproductive function and fertility (21, 35). Median values ranged from 2.7 to 13.75 mm, with most medians falling between 4.2 and 8.1 mm, consistent with the range of normal endometrial thickness previously reported in women of reproductive and perimenopausal age, supporting the external validity of these data for the Indonesian population. This pattern suggests that a uniform reference range can be applied across different ethnicities, while still acknowledging inter-individual variability.

Overall, the endometrial thickness measurements observed in this study exhibit significant consistency with established reference values from surrounding Asian populations. A study of 517 Malaysian midlife women aged 45 years revealed an average endometrial thickness of 8.05 mm in premenopausal women and 4.14 mm in postmenopausal women (36). Meanwhile, a Thai cohort reported similar premenopausal ranges of approximately 8.1 to 10.9 mm (37). A Japanese study has similarly revealed comparable premenopausal levels, though with slight differences possibly due to changes in the body mass index, the occurrence of irregular bleeding, and previous obstetric history (13). The results obtained from the current Indonesian cohort correspond with the published ranges in Southeast and East Asia, hence strengthening the external validity of our reference data. Significantly, direct statistical comparison among these populations was not feasible due to the variability in measurement techniques, study designs, and sonographic equipment utilized across studies.

The same biological process also drives angiogenesis to support tumor growth, deeper myometrial infiltration, and potential lymphovascular dissemination (38). Specifically myometrial invasion is directly linked to the prognostic profile of EC and constitutes a key criterion for stratifying endometrial tumors into low- and high-risk categories (39). This is reflected in the observed relationship between the depth of myometrial invasion and the color Doppler score, where higher invasion depth is associated with increased vascularization. Taken together, these findings indicate that variations in endometrial thickness are closely correlated with patient age and clinical condition, capturing both physiological aging-related changes and pathological transformation of the endometrium. Nevertheless, this study has several limitations. Data from endometrial cancer patients younger than 20 years were not available, precluding comparison of this age group with the normal cohort. In addition, this study primarily focused on the general comparison of endometrial thickness according to age between normal and cancer conditions; therefore, other potentially influential factors, such as metabolic status, were not analyzed and were assumed to be within normal conditions. Accordingly, we recommend that future studies incorporate these factors to provide a more comprehensive evaluation.

## 5 Conclusion

This study characterizes the average age at menopause in Indonesian women from diverse ethnic backgrounds and establishes population-specific reference values for normal endometrial thickness. Significant differences in endometrial thickness were observed between premenopausal and postmenopausal women, underscoring the influence of age and menopausal status on endometrial morphology. Furthermore, endometrial thickness was significantly greater in women with endometrial cancer and remained independently associated with malignancy after adjustment for age and body mass index. Age-specific cutoff values were also identified, supporting the clinical utility of endometrial thickness measurement in the evaluation of endometrial cancer. Collectively, these findings provide important reference data for Indonesian women and highlight the value of age-informed interpretation of endometrial thickness in both normal and malignant conditions. Nonetheless, the results must be understood within the framework of the retrospective study design. Additional prospective studies with bigger sample sizes are necessary to confirm these findings and enhance their generalizability.

## Data Availability

The original contributions presented in the study are included in the article/Supplementary Material, further inquiries can be directed to the corresponding author.
