# Supplementary material for: Endometrial thickness evaluation with ultrasonography assessment among normal and endometrial cancer across multi-ethnic Indonesian women
Source: Front Reprod Health. 2026 Jul 23;8:1826142. doi: 10.3389/frph.2026.1826142 (PMC13443303; doi:10.3389/frph.2026.1826142)
Supplement: Supplementary file 1 [file Table1.docx]

Supplementary Material

**Table S1.** Normal endometrium variations among ethnic group in Indonesia

| Ethnic group | n | Mean ± standard error (mm) | Kruskal-Wallis test value | p-value |
| --- | --- | --- | --- | --- |
| Acehnese | 2 | 8.10±1.70 | 13.821 |  |
| Balinese | 3 | 5.47±1.15 |  |  |
| Banjarese | 3 | 5.67±2.31 |  |  |
| Batak | 17 | 5.71±0.79 |  |  |
| Batavian | 33 | 6.02±0.56 |  |  |
| Buginese | 2 | 6.70±0.50 |  |  |
| Chinese | 15 | 5.47±1.32 |  |  |
| Javanese | 45 | 6.61±0.46 |  | 0.463 |
| Lampung | 2 | 13.75±1.05 |  |  |
| Malay | 8 | 5.85±1.16 |  |  |
| Minahasa | 2 | 6.30±3.10 |  |  |
| Minangkabau | 3 | 3.93±1.35 |  |  |
| Palembang | 2 | 5.30±0.01 |  |  |
| Sundanese | 30 | 6.46±0.73 |  |  |
| Toraja | 3 | 4.20±0.01 |  |  |

**Table S2.** Pairwise Mann-Whitney U comparison between age categories (normal endometrium)

| Age Group | <21 | 21-30 | 31-40 | 41-50 | 51-60 | >60 |
| --- | --- | --- | --- | --- | --- | --- |
| < 21 | - |  |  |  |  |  |
| 21-30 | 61.50 | - |  |  |  |  |
| 31-40 | 72.50 | 643.50 | - |  |  |  |
| 41-50 | 73.50 | 701.00 | 1283.50 | - |  |  |
| 51-60 | 16.00^*^ | 188.50^*^ | 351.00^*^ | 370.50^*^ | - |  |
| > 60 | 1.00^*^ | 18.50^*^ | 28.00^*^ | 30.50^*^ | 46.00 | - |

* *p* < 0.05

**Table S3**. Pairwise Mann-Whitney comparisons of ET between normal and cancer groups across age categories

| Age Group | U value | *p* value |
| --- | --- | --- |
| 21-30 | 17.000 | 0.006 |
| 31-40 | 199.000 | < 0.001 |
| 41-50 | 22.000 | < 0.001 |
| 51-60 | 6.000 | < 0.001 |
| > 60 | 0.000 | 0.001 |
